# Association between grip strength and major adverse cardiovascular events in patients with metabolic dysfunction-associated steatotic liver disease: a prospective cohort study in the UK Biobank

**DOI:** 10.3389/fendo.2026.1914934

**Published:** 2026-08-25

**Authors:** Yanxiao Xie, Ren Lin, Ziqi Zhang, LeShen Lian

**Affiliations:** 1 Department of Respiratory Medicine, Dongguan Hospital of Traditional Chinese Medicine, Dongguan, Guangdong, China; 2 The Ninth Clinical Medical College, Guangzhou University of Traditional Chinese Medicine, Dongguan, Guangdong, China; 3 Physical Examination Center, Renmin Hospital of Wuhan University, Wuhan, China; 4 Institute of Digestive Diseases, Longhua Hospital, Shanghai University of Traditional Chinese Medicine, Shanghai, China

**Keywords:** cardiovascular disease, grip strength, metabolic dysfunction-associated steatotic liver disease, muscle strength, UK Biobank

## Abstract

**Background:**

Cardiovascular disease represents the primary cause of mortality among individuals with metabolic dysfunction-associated steatotic liver disease (MASLD). We aimed to examine the association between grip strength and the risk of incident major adverse cardiovascular events (MACE) in this population.

**Methods:**

This prospective cohort analysis included participants with MASLD from the UK Biobank. Participants were categorized into high, medium, and low grip strength groups based on sex-specific tertiles. Cox proportional hazards models were used to estimate hazard ratios (HRs) and 95% confidence intervals (CIs) for the associations between grip strength and incident MACE and its components (heart failure, myocardial infarction, and stroke).

**Results:**

A total of 119, 004 participants with MASLD were included (mean [SD] age, 57.24 [7.89] years; 69, 421 men [58.3%]). During a median (IQR) follow-up of 13.6 (12.7-14.4) years, 14, 137 (11.9%) incident MACE cases were recorded. Compared with the high grip strength group, the low grip strength group exhibited a higher risk of MACE (HR = 1.35, 95% CI: 1.29-1.41, *P* < 0.001), heart failure (HR = 1.52, 95% CI: 1.42-1.62, *P* < 0.001), myocardial infarction (HR = 1.26, 95% CI: 1.19-1.34, *P* < 0.001), and stroke (HR = 1.33, 95% CI: 1.21-1.45, *P* < 0.001). A significant interaction between grip strength and sex was observed (*P* for interaction = 0.001), with the association between low grip strength and MACE being stronger in women (HR = 1.45, 95% CI: 1.34-1.56, *P* < 0.001) than in men (HR = 1.29, 95% CI: 1.16-1.45, *P* < 0.001). No significant interaction was observed for age.

**Conclusion:**

In individuals with MASLD, lower grip strength was associated with an increased risk of MACE, with a stronger association observed in women. These findings highlight grip strength as a potential marker of cardiovascular risk among individuals with MASLD.

## Introduction

Metabolic dysfunction-associated steatotic liver disease (MASLD), formerly known as non-alcoholic fatty liver disease (NAFLD), is the most prevalent chronic liver disorder globally, impacting about one-third of the global population (1). MASLD is closely linked to components of the metabolic syndrome, such as obesity, diabetes and dyslipidemia (2). These associated systemic metabolic disturbances not only drive liver-related morbidity but also substantially increase the incidence and mortality of cardiovascular disease (CVD). A meta-analysis by Mantovani et al. demonstrated that MASLD was associated with a 45% higher risk of fatal and nonfatal cardiovascular events (3). Notably, previous studies have shown that CVD has surpassed liver-related complications as the leading cause of death among patients with MASLD (4–6). Consequently, identifying simple, readily available and effective predictors of cardiovascular risk in this population is of considerable public health importance for enabling early intervention and improving prognosis.

As the largest metabolic organ, skeletal muscle plays an important role in glucose disposal, lipid metabolism, and the regulation of inflammation. In recent years, the concept of a “muscle-liver axis” has been proposed, highlighting a critical bidirectional interaction between muscle and liver health (7, 8). Current evidence supporting the muscle-liver axis has largely originated from mechanistic studies in animal models, demonstrating that skeletal muscle-derived myokines and metabolic alterations can influence hepatic inflammation and lipid metabolism (9). Human studies have further supported the clinical relevance of this bidirectional relationship (10, 11). Emerging evidence indicates that grip strength, a simple, objective, and cost-effective indicator of overall muscle function, is significantly associated with higher all-cause mortality, cardiovascular mortality, and the risk of multiple chronic diseases (12, 13). Although impaired muscle health has been associated with increased cardiovascular risk among individuals with MASLD, existing evidence has predominantly been derived from cross-sectional studies, and whether grip strength predicts future cardiovascular events in patients with MASLD remains unclear (14). Addressing this gap is crucial, as clarifying this relationship may not only deepen our understanding of the muscle-liver-cardiovascular axis but also offer a practical, non-invasive tool to support cardiovascular risk stratification in MASLD care.

Based on this rationale, we used large-scale prospective cohort data from the UK Biobank to investigate the association between baseline grip strength and the risk of incident major adverse cardiovascular events (MACE) and its individual components-heart failure (HF), myocardial infarction (MI), and stroke. We hypothesized that lower baseline grip strength is associated with a higher risk of incident MACE and its individual components. Additionally, we explored whether these associations were modified by age or sex.

## Methods

### Study population and design

This study was based on data from the UK Biobank, a large prospective population-based cohort comprising more than 500, 000 individuals aged 40–69 years, who were recruited between 2006 and 2010 from 22 assessment centers. The study collected extensive data, including detailed questionnaires, physical measurements, biological samples, and genetic information.

The diagnosis of MASLD was based on the currently recommended consensus criteria (15): (1) presence of hepatic steatosis, defined non-invasively in this study by a Fatty Liver Index (FLI) ≥ 60 (16). (2) Concurrent presence of at least one of the following cardiometabolic risk factors: body mass index (BMI) ≥ 25 kg/m² or elevated waist circumference (≥ 94 cm for men, ≥ 80 cm for women); blood pressure ≥ 130/85 mmHg or use of antihypertensive medication; fasting serum glucose ≥100 mg/dL or HbA1c ≥5.7% or use of glucose-lowering medication; triglycerides (TG) ≥ 150 mg/dL or use of lipid-lowering medication; or reduced high-density lipoprotein cholesterol (HDL-C) (< 40 mg/dL for men, < 50 mg/dL for women). We sequentially excluded individuals with excessive alcohol consumption (>30 g/day for men and >20 g/day for women), pregnant women, individuals with other known liver diseases (including chronic viral hepatitis [ICD-9: 070, 5731; ICD-10: B18-B19], alcoholic liver disease [ICD-9: 5710-5713; ICD-10: K70], toxic liver disease [ICD-10: K71], Wilson’s disease [ICD-10: E83.0], hemochromatosis [ICD-9: 2750; ICD-10: E83.1], and autoimmune hepatitis [ICD-9: 5716; ICD-10: K74.3, K74.5, K75.4]), participants who experienced MACE at baseline, those lacking grip strength or other covariate data, and participants who withdrew their informed consent. Consequently, a total of 119, 004 patients with MASLD were retained for the final analysis (**Supplementary Figure 1**). The UK Biobank protocol was approved by the ethics committee, and all participants provided written informed consent. This study was conducted in accordance with the Declaration of Helsinki.

### Assessment of grip strength

Grip strength was assessed at baseline using a Jamar J00105 hydraulic hand dynamometer. Measurements were obtained from both hands, and the mean of left- and right-hand grip strength values was used in the present study. To account for sex differences and improve clinical interpretability, grip strength was treated as a categorical variable, stratified into low, medium, and high groups based on sex-specific tertiles. Among women, the tertile cut-off values were 0.5-20.0 kg (lowest tertile), 20.5-25.0 kg (middle tertile), and 25.5-54.0 kg (highest tertile). Among men, the corresponding tertile categories were 1.0-35.5 kg, 36.0-43.0 kg, and 43.5-85.0 kg, respectively.

### Ascertainment of MACE

MACE were defined as a composite outcome including cardiovascular mortality (ICD-10 codes I10, I11, I13, I20-I51, I60-I69), hospitalization for myocardial infarction (ICD-10 codes I21, I22, I23, I24.1, I25.2), heart failure (ICD-10 code I50), or stroke (ICD-10 codes I60, I61, I63, I64), identified using relevant ICD-10 codes. Outcome ascertainment was achieved through linkage with national hospital and mortality databases across England, Scotland, and Wales. Participants were followed from the baseline assessment until the occurrence of a first MACE event, death, loss to follow-up, or the end of follow-up (November 30, 2022), whichever occurred earliest.

### Statistical analysis

Continuous variables are summarized as mean with standard deviation (SD), and categorical variables as frequency (percentage). Associations between grip strength levels and the risk of MACE were examined using Cox proportional hazards models, with effect estimates presented as HRs and their 95% CIs. The multivariable models were adjusted for the following covariates: age (continuous), sex, race/ethnicity, education level, smoking status, alcohol intake, body mass index (BMI; continuous), Townsend Deprivation Index (continuous), hypertension, type 2 diabetes, any cancer, and sleep duration (continuous). The proportional hazards assumption was evaluated using Schoenfeld residuals and was found to be satisfied (**Supplementary Table 1**). P for trend was calculated by modeling the grip strength tertiles as an ordinal variable in the regression models. Restricted cubic splines (RCS) with three knots (placed at the 10th, 50th, and 90th percentiles of grip strength distribution) were fitted within the Cox models to visualize the dose-response relationship between grip strength and the risk of MACE and to test for potential non-linearity. Three knots were chosen as the minimum number required to detect potential non-linear relationships while avoiding overfitting, consistent with standard practice in large epidemiological studies. Non-linearity was tested using a likelihood ratio test comparing the linear and spline models. Predefined subgroup analyses were conducted according to age (<60/≥60 years) and sex (male/female), and the significance of interaction was evaluated by introducing multiplicative interaction terms into the models. Several sensitivity analyses were conducted to assess the robustness of our findings. First, participants who developed MACE within the initial two years of follow-up were excluded to reduce the potential influence of reverse causation. Second, we accounted for the competing risk of non-cardiovascular death by fitting Fine-Gray competing risk models for incident MACE. Third, we repeated the analyses after excluding individuals with extreme grip strength values, defined as those lying more than 2.5 SD above or below the sex-specific mean. Fourth, to address missing data on covariates, we performed multiple imputation by chained equations. Fifth, in an additional fully adjusted model, we further adjusted for C-reactive protein (CRP) and LDL-C. Sixth, we additionally adjusted for physical activity status. Physical activity was categorized according to whether participants met the recommended moderate-to-vigorous physical activity level, defined as achieving at least 150 minutes/week of moderate activity or 75 minutes/week of vigorous activity. Seventh, we repeated the analyses using BMI-normalized grip strength (grip strength/BMI) as the exposure variable. All analyses were conducted using R software (version 4.2.3), and statistical significance was defined as a two-sided P value <0.05.

## Results

**Table 1** summarizes the baseline characteristics of all included participants. The mean (SD) age was 57.24 (7.89) years, with 69, 421 (58.3%) men and 49, 583 (41.7%) women. Compared to the high grip strength group, participants in the low grip strength group were generally older (59.38 vs. 54.46 years), had lower educational attainment, a higher Townsend deprivation index, and lower alcohol intake. They also exhibited a higher prevalence of hypertension (71.7% vs. 64.5%), type 2 diabetes (13.4% vs. 6.5%), and any cancer (10.5% vs. 7.3%).

**Table 1:** Baseline characteristics of participants according to grip strength.

Characteristic	Overall N = 119, 004	Grip strength			
		High N = 38, 076	Medium N = 38, 797	Low N = 42, 131	P-value
Age, years	57.24 (7.89)	54.46 (7.88)	57.64 (7.67)	59.38 (7.34)	<0.001
Sex					<0.001
Female	49, 583.0 (41.7%)	15, 656.0 (41.1%)	15, 031.0 (38.7%)	18, 896.0 (44.9%)	
Male	69, 421.0 (58.3%)	22, 420.0 (58.9%)	23, 766.0 (61.3%)	23, 235.0 (55.1%)	
Race and ethnicity					<0.001
White	111, 478.0 (93.7%)	36, 040.0 (94.7%)	36, 724.0 (94.7%)	38, 714.0 (91.9%)	
Others	7, 526.0 (6.3%)	2, 036.0 (5.3%)	2, 073.0 (5.3%)	3, 417.0 (8.1%)	
Educational level					<0.001
<High school	46, 449.0 (39.0%)	12, 171.0 (32.0%)	14, 891.0 (38.4%)	19, 387.0 (46.0%)	
High school	5, 924.0 (5.0%)	1, 993.0 (5.2%)	1, 928.0 (5.0%)	2, 003.0 (4.8%)	
College or above	66, 631.0 (56.0%)	23, 912.0 (62.8%)	21, 978.0 (56.6%)	20, 741.0 (49.2%)	
Townsend deprivation index	-1.03 (3.20)	-1.31 (3.08)	-1.18 (3.15)	-0.63 (3.33)	<0.001
BMI, kg/m^2^	31.78 (4.63)	31.91 (4.60)	31.50 (4.47)	31.93 (4.77)	<0.001
Smoking status					<0.001
Never	64, 093.0 (53.9%)	20, 928.0 (55.0%)	20, 702.0 (53.4%)	22, 463.0 (53.3%)	
Former	43, 033.0 (36.2%)	13, 199.0 (34.7%)	14, 234.0 (36.7%)	15, 600.0 (37.0%)	
Current	11, 878.0 (10.0%)	3, 949.0 (10.4%)	3, 861.0 (10.0%)	4, 068.0 (9.7%)	
Alcohol consumption	59.39 (62.78)	62.98 (63.43)	62.47 (63.50)	53.30 (61.04)	<0.001
Sleep duration, hours/day	7.12 (1.21)	7.09 (1.10)	7.12 (1.16)	7.15 (1.33)	<0.001
DBP, mmHg	85.28 (9.64)	86.30 (9.51)	85.41 (9.55)	84.26 (9.74)	<0.001
SBP, mmHg	141.73 (17.43)	141.30 (16.93)	142.30 (17.51)	141.59 (17.76)	<0.001
Fasting glucose, mmol/L	5.35 (1.63)	5.21 (1.39)	5.33 (1.60)	5.50 (1.84)	<0.001
HbA1c, %	5.65 (0.79)	5.55 (0.69)	5.64 (0.78)	5.76 (0.87)	<0.001
TG, mmol/L	2.40 (1.18)	2.40 (1.19)	2.41 (1.18)	2.40 (1.18)	0.169
TC, mmol/L	5.70 (1.22)	5.79 (1.16)	5.71 (1.21)	5.60 (1.26)	<0.001
HDL-c, mmol/L	1.22 (0.28)	1.21 (0.27)	1.22 (0.28)	1.23 (0.29)	<0.001
LDL-c, mmol/L	3.64 (0.92)	3.73 (0.88)	3.65 (0.91)	3.56 (0.94)	<0.001
Hypertension					<0.001
Yes	81, 345.0 (68.4%)	24, 574.0 (64.5%)	26, 576.0 (68.5%)	30, 195.0 (71.7%)	
No	37, 659.0 (31.6%)	13, 502.0 (35.5%)	12, 221.0 (31.5%)	11, 936.0 (28.3%)	
Type 2 diabetes					<0.001
Yes	11, 677.0 (9.8%)	2, 493.0 (6.5%)	3, 538.0 (9.1%)	5, 646.0 (13.4%)	
No	107, 327.0 (90.2%)	35, 583.0 (93.5%)	35, 259.0 (90.9%)	36, 485.0 (86.6%)	
Any cancer					<0.001
Yes	10, 722.0 (9.0%)	2, 780.0 (7.3%)	3, 538.0 (9.1%)	4, 404.0 (10.5%)	
No	108, 282.0 (91.0%)	35, 296.0 (92.7%)	35, 259.0 (90.9%)	37, 727.0 (89.5%)	

For continuous variables, data were described as mean (SD); For categorical variables, data were described as frequencies (percentages).

Over a median follow-up of 13.6 years (IQR, 12.7-14.4 years), 14, 137 participants experienced at least one MACE event. In separate analyses of individual cardiovascular outcomes, 6, 214 participants experienced HF, 7, 360 experienced MI, and 3, 500 experienced stroke. Kaplan-Meier curves illustrated the cumulative incidence of events across the grip strength categories. For all endpoints-MACE, HF, MI, and stroke-the cumulative incidence was highest in the low grip strength group, intermediate in the medium group, and lowest in the high group (P_log-rank_ < 0.001; **Supplementary Figure 2**).

**Figure 1:**
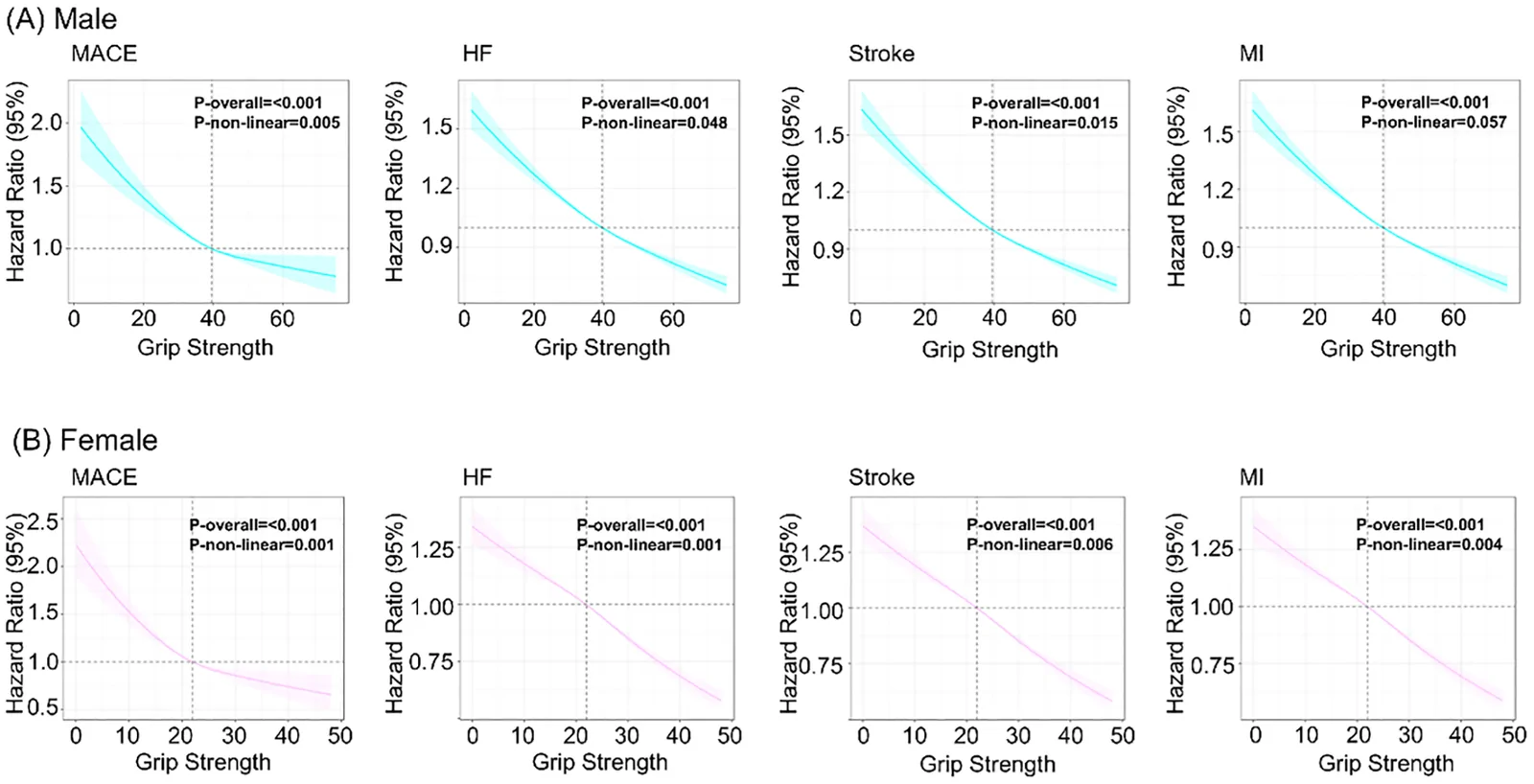
Restricted cubic spline plot of the association between grip strength and MACE risk stratified by sex. **(A)** Male; **(B)** Female. The model was adjusted for age, race/ethnicity, education, smoking status, alcohol intake, body mass index, Townsend deprivation index, hypertension, type 2 diabetes, any cancer, and sleep duration.

In restricted cubic spline analyses, nonlinear associations between grip strength and all cardiovascular outcomes were observed (P-nonlinearity < 0.05), except for MI in men (**Figure 1**). In the univariable model, compared with the high grip strength group, the low grip strength group was associated with a significantly increased risk of MACE (HR = 2.00, 95% CI: 1.92-2.09), followed by the medium group (HR = 1.40, 95% CI: 1.34-1.47, **Table 2**). After adjustment for relevant covariates, these associations remained significant (low vs. high: HR = 1.35, 95% CI: 1.29-1.41; medium vs. high: HR = 1.10, 95% CI: 1.05-1.15), indicating that lower grip strength was an independent risk factor for MACE. However, the noticeable decrease in effect estimates suggests that a significant portion of the observed risk was driven by underlying confounding variables. In analyses of specific MACE components, lower grip strength was associated with higher risks of HF (HR = 1.52, 95% CI: 1.42-1.62), MI (HR = 1.26, 95% CI: 1.19-1.34), and stroke (HR = 1.33, 95% CI: 1.21-1.45) after multivariable adjustment. Participants in the medium grip strength group also had a moderately increased risk of HF (HR = 1.18, 95% CI: 1.09-1.26) and stroke (HR = 1.14, 95% CI: 1.04-1.25) compared with those in the high grip strength group. For MI, the association for the medium grip strength group was attenuated after multivariable adjustment and was no longer statistically significant (HR, 1.05; 95% CI, 0.99-1.12), suggesting a less consistent gradient across grip strength categories for this outcome.

**Table 2:** Associations between grip strength and MACE in MASLD patients.

Outcome	Hazard ratio (95% CI)			P-value for trend
	High	Medium	Low	
MACE				
No of events	3202/38076	4417/38797	6518/42131	
Model 1	1.00 (Ref)	1.40 (1.34-1.47)	2.00 (1.92-2.09)	<0.001
Model 2	1.00 (Ref)	1.10 (1.05-1.15)	1.35 (1.29-1.41)	<0.001
HF				
No of events	1198/38076	1878/38797	3138/42131	
Model 1	1.00 (Ref)	1.59 (1.48-1.71)	2.56 (2.39-2.74)	<0.001
Model 2	1.00 (Ref)	1.18 (1.09-1.26)	1.52 (1.42-1.62)	<0.001
MI				
No of events	1782/38076	2321/38797	3257/42131	
Model 1	1.00 (Ref)	1.31 (1.23-1.40)	1.76 (1.66-1.86)	<0.001
Model 2	1.00 (Ref)	1.05 (0.99-1.12)	1.26 (1.19-1.34)	<0.001
Stroke				
No of events	777/38076	1126/38797	1597/42131	
Model 1	1.00 (Ref)	1.46 (1.33-1.60)	1.97 (1.81-2.15)	<0.001
Model 2	1.00 (Ref)	1.14 (1.04-1.25)	1.33 (1.21-1.45)	<0.001

Model 1: unadjusted for covariates.

Model 2: adjusted for age, sex, education, ethnicity, Townsend Deprivation Index, body mass index, smoking status, alcohol consumption, hypertension, type 2 diabetes, any cancer and sleep duration.

**Figure 2:**
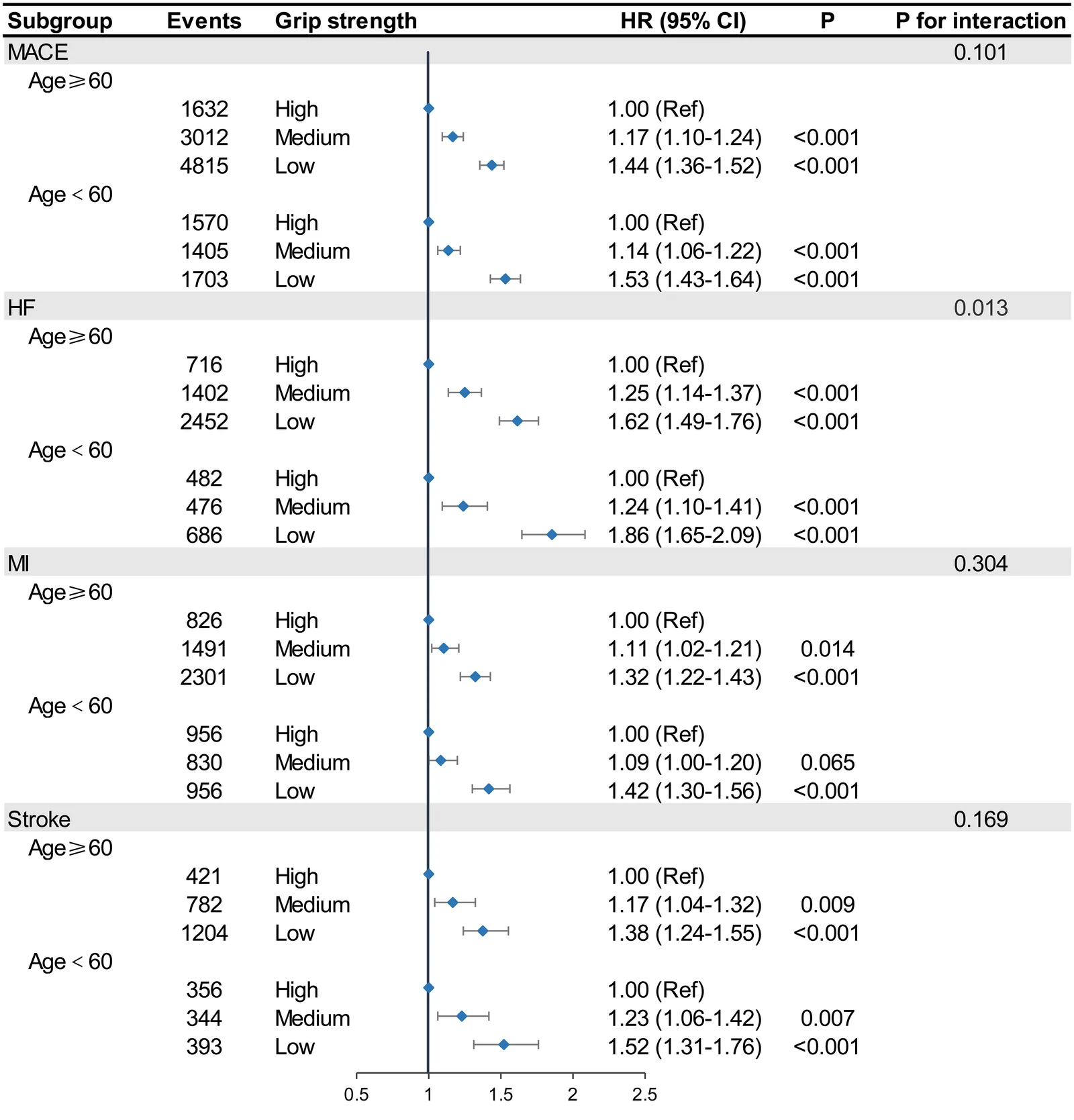
Subgroup analysis of the association between grip strength and MACE by age. The forest plot presents the multivariable-adjusted HRs and 95% CIs for the associations between grip strength levels and the risks of MACE, HF, MI, and stroke in the age subgroups (<60 years and ≥60 years). All models were fully adjusted for potential confounders. The *P* for interaction was set to assess the significance of the interaction effect between age and grip strength categories.

**Figure 3:**
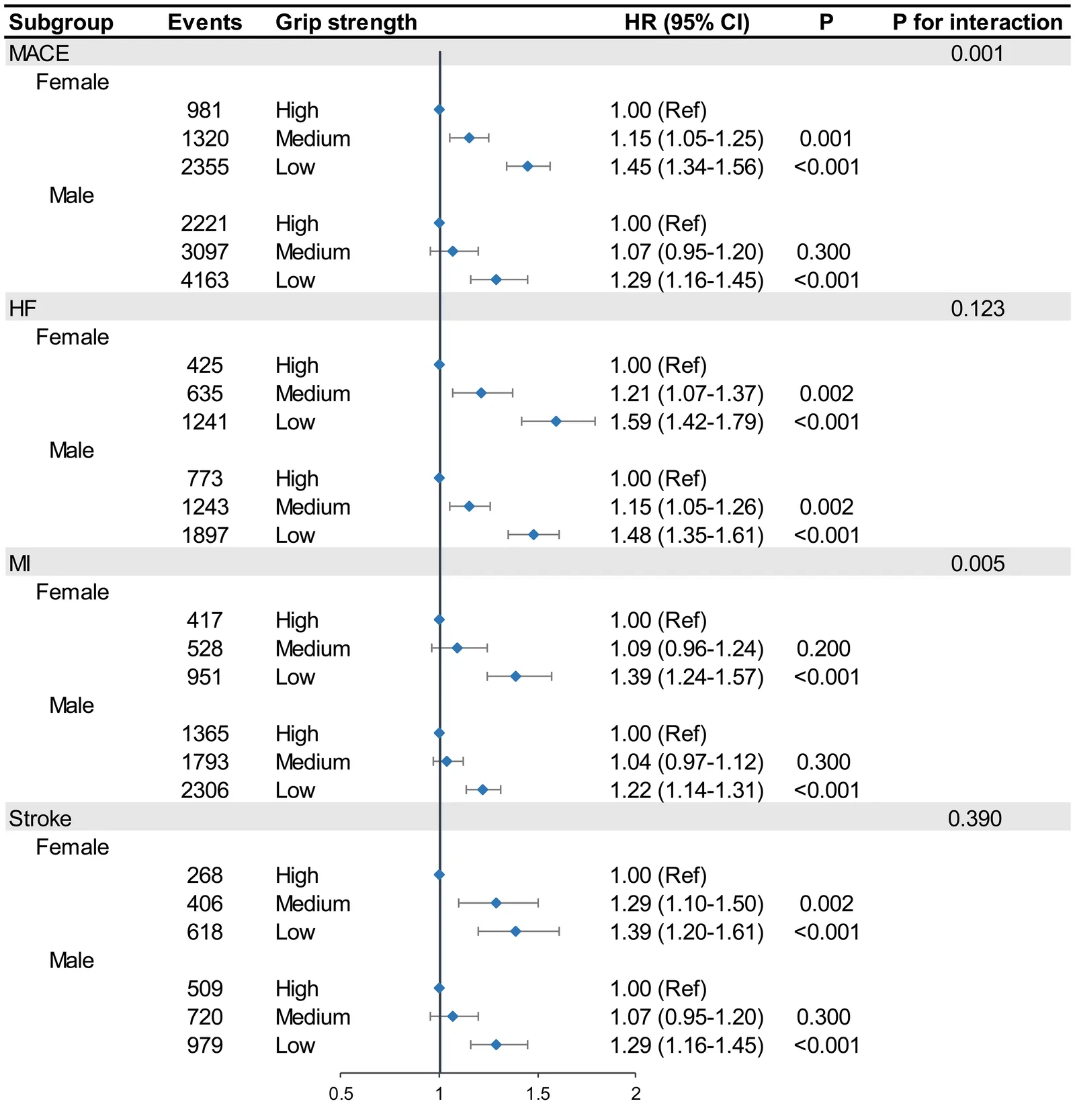
Subgroup analysis of the association between grip strength and MACE by sex. The forest plot presents the multivariable-adjusted HRs and 95% CIs for the associations between grip strength levels and the risks of MACE, HF, MI, and stroke in male and female subgroups. All models were fully adjusted for potential confounders. The *P* for interaction was set to assess the significance of the interaction effect between sex and grip strength categories.

In age subgroup analyses (**Figure 2**), no significant interaction with age was observed for MACE, MI, or stroke (P for interaction >0.05). However, for the outcome of HF, a significant interaction with age was observed (P for interaction = 0.013), indicating that the excess risk associated with low grip strength was more pronounced in the younger group (<60 years; HR _low vs. high_ = 1.86, 95% CI: 1.65-2.09) than in the older group (HR _low vs. high_ = 1.62, 95% CI: 1.49-1.76). In sex subgroup analyses (**Figure 3**), low grip strength showed a stronger association with MACE in women (HR _low vs. high_ = 1.45, 95% CI: 1.34-1.56) than in men (HR _low vs. high_ = 1.29, 95% CI: 1.16-1.45), with a significant interaction by sex (P for interaction = 0.001). A significant grip strength-sex interaction was also observed for MI (P for interaction = 0.005), with the excess risk associated with low grip strength being greater in women (HR _low vs. high_ = 1.39, 95% CI: 1.24-1.57) than in men (HR _low vs. high_ = 1.22, 95% CI: 1.14-1.31).

In sensitivity analyses, the findings were broadly consistent with those of the primary models. Excluding participants who experienced MACE within the initial 2 years of follow-up (**Supplementary Table 2**), using the Fine-Gray competing risk model (**Supplementary Table 3**), and removing individuals with extreme grip strength values (**Supplementary Table 4**) all yielded similar associations between lower grip strength and increased risks of MACE and its individual components. Further adjustment for LDL-C and CRP did not alter these findings (**Supplementary Table 5**). In the multiple imputation analyses, the estimated effect sizes for the associations between low grip strength and MACE were modestly stronger (**Supplementary Table 6**); compared with high grip strength, medium grip strength was also associated with a modestly higher risk of all endpoints, including MI; after additional adjustment for physical activity status, the associations remained significant for MACE (HR _low vs. high_ =1.35, 95%CI: 1.28–1.42; HR _medium vs. high_=1.09, 95%CI: 1.03–1.15) (**Supplementary Table 7**). Similarly, using BMI-normalized grip strength as the exposure, lower relative grip strength was associated with higher risks of MACE (HR _low vs. high_=1.77, 95%CI: 1.67–1.88; HR _medium vs. high_=1.28, 95%CI: 1.23–1.33) (**Supplementary Table 8**). These analyses support the robustness of our main results.

## Discussion

In this large-scale prospective cohort study, we demonstrated that lower grip strength was independently associated with a higher risk of MACE, HF, MI, and stroke among patients with MASLD. These associations remained robust after adjustment for lifestyle factors and other covariates. Notably, low grip strength was more strongly associated with MACE in women than in men, highlighting the sex-specificity of grip strength as both a cardiovascular risk marker and a potential target for intervention. Taken together, these findings highlight grip strength as a potential marker of cardiovascular risk in individuals with MASLD, with a stronger association observed among women.

Our findings are broadly consistent with previous research conducted in both general and disease-specific populations. In a prospective cohort study of 5, 300 community-dwelling adults in the UK, trajectory analyses demonstrated that individuals with consistently low grip strength had more than a twofold higher risk of CVD, with significantly elevated risks of MI, angina, stroke, and HF (17). Similarly, an analysis of the US National Health and Nutrition Examination Survey showed that adults with low grip strength had a significantly higher risk of all-cause and CVD mortality, irrespective of sex (18). Our study extends this association to the specific high-risk population of individuals with MASLD, further underscoring the broad importance of muscular function in cardiovascular health. The association between grip strength and MACE remained significant even after adjustment for various traditional cardiovascular risk factors, including BMI, suggesting that grip strength may provide independent predictive value beyond conventional risk factors. By dissecting the composite MACE endpoint, we show that the association with grip strength is not driven by any single cardiovascular outcome. Given that grip strength measurement is simple, quick, and low-cost, it has the potential to be implemented in primary care and resource-limited settings. However, its widespread adoption may still depend on the availability of hand dynamometers and local healthcare resources. If further validated, grip strength may serve as a pragmatic marker for cardiovascular risk assessment in individuals with MASLD.

Subgroup analyses in our study revealed that the association between low grip strength and HF risk was significantly stronger in relatively younger MASLD patients (aged <60 years) compared to their older counterparts (≥60 years). Consistent with our findings, a previous UK Biobank study also suggested that the association between grip strength and cardiovascular outcomes was more pronounced among younger individuals (19). Grip strength serves not merely as an indicator of upper limb muscle power but as a comprehensive biomarker of neuromuscular control, metabolic health, and functional reserve (20, 21). In younger MASLD patients (<60 years), diminished grip strength may signal early systemic functional decline, suggesting their physiological reserve is already compromised. This renders the cardiovascular system more vulnerable to metabolic stress imposed by MASLD, potentially leading to decompensation and HF. Conversely, in older patients (≥60 years), some degree of muscle loss and functional decline represents an expected age-related change, potentially weakening its signal strength as an abnormal risk indicator. This finding suggests that grip assessment holds particular early warning value in younger MASLD patients, where detected weakness should be considered a strong risk signal. Future studies should longitudinally track grip strength trajectories to more precisely elucidate its role in HF prevention among MASLD patients.

Our study also found a stronger association between grip strength and MACE in female patients with MASLD. Several factors may explain this observation. First, from a biological perspective, women have significantly lower muscle mass than men (22), thus a relative loss of muscle function may exert a greater impact on their metabolic and cardiovascular systems. Second, endocrine factors play an important role; the decline in estrogen levels following menopause is associated with both sarcopenia and increased cardiovascular risk, potentially making grip strength a more sensitive risk indicator in women (23). Furthermore, our study also revealed a stronger association between grip strength and MI in women, which likely contributed to the overall stronger association with MACE. This finding suggests that in clinical practice, the assessment of muscle strength in female MASLD patients warrants particular attention. Future sex-specific research should delve deeper into the mechanisms underlying this disparity.

The association between grip strength and MACE is likely mediated through several interconnected pathophysiological pathways. Firstly, skeletal muscle is a primary determinant of insulin sensitivity. Low grip strength, reflecting diminished muscle mass and function, can exacerbate insulin resistance (9, 24), subsequently promoting hyperglycaemia, hyperinsulinaemia, and dyslipidaemia—shared pathological features of both MASLD and MACE. Secondly, skeletal muscle functions as an endocrine organ, secreting various myokines—such as irisin, IL-6, and IL-15—that systemically regulate inflammation, metabolism, and cardiovascular function (25, 26). Low grip strength may signify reduced beneficial myokine secretion, fostering a chronic low-grade inflammatory state that can accelerate vascular endothelial injury and promote atherosclerosis, thereby increasing MACE risk. Thirdly, sarcopenia often coexists with increased visceral adiposity, a condition termed “myosteatosis.” This adverse body composition profile is associated with more severe metabolic dysfunction and elevated cardiovascular risk (27, 28). Finally, low grip strength was associated with a range of adverse characteristics, including older age, unfavorable metabolic profiles, and a higher burden of comorbidities, all of which are established risk factors for MACE. Although we adjusted for multiple cardiovascular and lifestyle-related factors, residual confounding cannot be completely excluded. In particular, physical activity represents an important potential confounder, as higher levels of physical activity may contribute to preserved muscle strength while independently reducing cardiovascular risk. However, grip strength reflects not only habitual physical activity but also broader aspects of musculoskeletal health, including muscle function, neuromuscular integrity, and physiological reserve. In our sensitivity analysis, additional adjustment for physical activity resulted in minimal changes in the observed associations, suggesting that the relationship between lower grip strength and MACE was unlikely to be fully explained by differences in physical activity levels. Although resistance training has been shown to improve muscle strength and cardiovascular health in the general population (29), future randomized controlled trials are warranted to determine whether interventions aimed at improving muscle strength, including structured exercise programs and targeted nutritional strategies, can effectively reduce cardiovascular risk in individuals with MASLD.

The strengths of this study include its large-scale, prospective design and long follow-up period, which enabled the documentation of a substantial number of MACE events. The availability of extensive data on covariates allowed for comprehensive adjustment, thereby helping to reduce potential confounding. However, several limitations should be considered. First, the diagnosis of MASLD was based on the FLI rather than the gold-standard liver biopsy, which may lead to misclassification. Nevertheless, the FLI has been validated as a reliable non-invasive tool for screening hepatic steatosis in population-based studies. Future validation studies in cohorts with imaging-based MASLD are needed to confirm the generalizability of our findings. Second, due to the observational nature of the study, causality cannot be inferred. Third, dietary data, such as total energy and protein intake, were only available for a subset of UK Biobank participants. Consequently, we were unable to adjust for these factors, even though they might be relevant. Fourth, multiple component and subgroup analyses were conducted without formal adjustment for multiple comparisons. Therefore, these findings should be considered exploratory and interpreted with caution. Fifth, given that most participants in the UK Biobank are White, and that the prevalence of MASLD, grip strength, and cardiovascular risk profiles may differ across racial and ethnic groups, caution is warranted when generalizing our findings to other populations. Sixth, the subgroup findings should be interpreted cautiously, as interaction analyses generally have limited statistical power. These findings may be subject to chance findings and require confirmation in independent cohorts. Seventh, grip strength was measured only at baseline, which may have introduced measurement error and regression dilution bias. Finally, although we performed a sensitivity analysis excluding participants who developed MACE within the first two years of follow-up, reverse causation cannot be completely excluded. Future studies are warranted to validate these results in more diverse racial, ethnic, and geographical populations.

## Conclusion

Utilizing large-scale prospective data from the UK Biobank, we found that low grip strength was independently associated with an increased risk of incident MACE and its individual components in patients with MASLD, with a more pronounced association in women. Future studies are needed to determine whether improving muscle strength and function can effectively reduce cardiovascular risk in MASLD and to clarify potential sex-specific effects.

## Data Availability

The original contributions presented in the study are included in the article/**Supplementary Material**. Further inquiries can be directed to the corresponding authors.
