# Supplementary material for: Association between grip strength and major adverse cardiovascular events in patients with metabolic dysfunction-associated steatotic liver disease: a prospective cohort study in the UK Biobank
Source: Front Endocrinol (Lausanne). 2026 Aug 25;17:1914934. doi: 10.3389/fendo.2026.1914934 (PMC13549879; doi:10.3389/fendo.2026.1914934)
Supplement: Supplementary file 1 [file SupplementaryFile1.docx]

**Supplementary Figure 1.** Study flow chart. Abbreviations: FLI, Fatty Liver Index; MACE, Major adverse cardiovascular events; MASLD, Metabolic dysfunction-associated steatotic liver disease.

**Supplementary Figure 2. Kaplan–Meier curves showing the cumulative incidence of (A) major adverse cardiovascular events, (B) heart failure, (C) myocardial infarction, and (D) stroke among individuals with metabolic dysfunction-associated steatotic liver disease, stratified by grip strength levels.** The total numbers of events were 14,137 for MACE, 6,214 for HF, 7,360 for MI, and 3,500 for stroke. The table below each panel displays the number of participants at risk at selected time points during follow-up.

**Supplementary Table 1**. Assessment of the proportional hazards assumption using Schoenfeld residuals.

| Variable | chisq | p |
| --- | --- | --- |
| Age | 2.293 | 0.130 |
| Sex | 65.225 | <0.001 |
| Ethnicity | 0.197 | 0.657 |
| Education level | 1.767 | 0.413 |
| BMI | 20.721 | <0.001 |
| TDI | 0.154 | 0.695 |
| Smoking status | 14.129 | 0.001 |
| Alcohol consumption | 0.770 | 0.380 |
| Sleep duration | 4.127 | 0.042 |
| Hypertension | 6.234 | 0.013 |
| T2D | 0.332 | 0.564 |
| Any cancer | 0.255 | 0.613 |
| Grip strength | 1.340 | 0.512 |

**Supplementary Table 2.** Associations between grip strength and incident MACE in MASLD patients after excluding participants with outcome events occurring within the first 2 years of follow-up.

| **Outcome** | **Hazard ratio (95% CI)** | | | **P-value for trend** |
| --- | --- | --- | --- | --- |
|  | **High** | **Medium** | **Low** |  |
| MACE | | | | |
| Model 1 | 1.00 (Ref) | 1.41 (1.34-1.47) | 1.99 (1.91-2.08) | <0.001 |
| Model 2 | 1.00 (Ref) | 1.10 (1.05-1.15) | 1.34 (1.28-1.40) | <0.001 |
| HF | | | | |
| Model 1 | 1.00 (Ref) | 1.57 (1.46-1.69) | 2.53 (2.37-2.72) | <0.001 |
| Model 2 | 1.00 (Ref) | 1.16 (1.07-1.25) | 1.50 (1.40-1.61) | <0.001 |
| MI | | | | |
| Model 1 | 1.00 (Ref) | 1.31 (1.23-1.39) | 1.74 (1.64-1.85) | <0.001 |
| Model 2 | 1.00 (Ref) | 1.05 (0.98-1.12) | 1.25 (1.17-1.33) | <0.001 |
| Stroke | | | | |
| Model 1 | 1.00 (Ref) | 1.47 (1.33-1.61) | 1.94 (1.77-2.12) | <0.001 |
| Model 2 | 1.00 (Ref) | 1.14 (1.04-1.26) | 1.30 (1.19-1.43) | <0.001 |

Model 1: unadjusted for covariates

Model 2: adjusted for age, sex, education, ethnicity, Townsend Deprivation Index, body mass index, smoking status, alcohol consumption, hypertension, type 2 diabetes, cancer and sleep duration.

**Supplementary Table 3.** Associations between grip strength and incident MACE in MASLD patients using a competing risk model

| **Outcome** | **Hazard ratio (95% CI)** | | | **P-value for trend** |
| --- | --- | --- | --- | --- |
|  | **High** | **Medium** | **Low** |  |
| MACE | | | | |
| Model 1 | 1.00 (Ref) | 1.39 (1.33-1.45) | 1.95 (1.87-2.03) | <0.001 |
| Model 2 | 1.00 (Ref) | 1.10 (1.05-1.15) | 1.33 (1.27-1.39) | <0.001 |
| HF | | | | |
| Model 1 | 1.00 (Ref) | 1.57 (1.46-1.69) | 2.48 (2.32-2.65) | <0.001 |
| Model 2 | 1.00 (Ref) | 1.17 (1.09-1.26) | 1.48 (1.38-1.59) | <0.001 |
| MI | | | | |
| Model 1 | 1.00 (Ref) | 1.30 (1.22-1.39) | 1.71 (1.60-1.82) | <0.001 |
| Model 2 | 1.00 (Ref) | 1.05 (0.98-1.13) | 1.24 (1.16-1.33) | <0.001 |
| Stroke | | | | |
| Model 1 | 1.00 (Ref) | 1.44 (1.32-1.58) | 1.91 (1.75-2.08) | <0.001 |
| Model 2 | 1.00 (Ref) | 1.14 (1.04-1.25) | 1.30 (1.19-1.42) | <0.001 |

Model 1: unadjusted for covariates

Model 2: adjusted for age, sex, education, ethnicity, Townsend Deprivation Index, body mass index, smoking status, alcohol consumption, hypertension, type 2 diabetes, cancer and sleep duration.

**Supplementary Table 4.** Associations between grip strength and incident MACE in MASLD patients after excluding individuals whose grip strength falls outside the range of mean ± 2.5 SD

| **Outcome** | **Hazard ratio (95% CI)** | | | **P-value for trend** |
| --- | --- | --- | --- | --- |
|  | **High** | **Medium** | **Low** |  |
| MACE | | | | |
| Model 1 | 1.00 (Ref) | 1.39 (1.33-1.46) | 1.96 (1.88-2.05) | <0.001 |
| Model 2 | 1.00 (Ref) | 1.10 (1.05-1.15) | 1.34 (1.28-1.40) | <0.001 |
| HF | | | | |
| Model 1 | 1.00 (Ref) | 1.58 (1.47-1.70) | 2.50 (2.34-2.67) | <0.001 |
| Model 2 | 1.00 (Ref) | 1.18 (1.09-1.27) | 1.50 (1.40-1.61) | <0.001 |
| MI | | | | |
| Model 1 | 1.00 (Ref) | 1.31 (1.23-1.39) | 1.72 (1.63-1.83) | <0.001 |
| Model 2 | 1.00 (Ref) | 1.05 (0.99-1.12) | 1.25 (1.18-1.33) | <0.001 |
| Stroke | | | | |
| Model 1 | 1.00 (Ref) | 1.45 (1.32-1.59) | 1.94 (1.78-2.12) | <0.001 |
| Model 2 | 1.00 (Ref) | 1.14 (1.04-1.25) | 1.32 (1.21-1.44) | <0.001 |

Model 1: unadjusted for covariates

Model 2: adjusted for age, sex, education, ethnicity, Townsend Deprivation Index, body mass index, smoking status, alcohol consumption, hypertension, type 2 diabetes, cancer and sleep duration.

**Supplementary Table 5.** Associations between grip strength and incident MACE in MASLD patients after further adjusting for LDL-C and CRP

| **Outcome** | **Hazard ratio (95% CI)** | | | **P-value for trend** |
| --- | --- | --- | --- | --- |
|  | **High** | **Medium** | **Low** |  |
| MACE | | | | |
| Model 1 | 1.00 (Ref) | 1.10 (1.05-1.15) | 1.35 (1.29-1.41) | <0.001 |
| Model 2 | 1.00 (Ref) | 1.09 (1.04-1.14) | 1.33 (1.27-1.39) | <0.001 |
| HF | | | | |
| Model 1 | 1.00 (Ref) | 1.18 (1.09-1.26) | 1.52 (1.41-1.62) | <0.001 |
| Model 2 | 1.00 (Ref) | 1.16 (1.08-1.25) | 1.48 (1.38-1.58) | <0.001 |
| MI | | | | |
| Model 1 | 1.00 (Ref) | 1.05 (0.99-1.12) | 1.26 (1.19-1.34) | <0.001 |
| Model 2 | 1.00 (Ref) | 1.05 (0.98-1.11) | 1.25 (1.18-1.33) | <0.001 |
| Stroke | | | | |
| Model 1 | 1.00 (Ref) | 1.14 (1.04-1.25) | 1.33 (1.21-1.45) | <0.001 |
| Model 2 | 1.00 (Ref) | 1.13 (1.03-1.24) | 1.32 (1.20-1.44) | <0.001 |

Model 1: adjusted for age, sex, education, ethnicity, Townsend Deprivation Index, body mass index, smoking status, alcohol consumption, hypertension, type 2 diabetes, cancer and sleep duration.

Model 2: adjusted for age, sex, education, ethnicity, Townsend Deprivation Index, body mass index, smoking status, alcohol consumption, hypertension, type 2 diabetes, cancer, sleep duration, LDL-C and CRP.

**Supplementary Table 6.** Associations between grip strength and incident MACE in MASLD patients after multiple imputation

| **Outcome** | **Hazard ratio (95% CI)** | | | **P-value for trend** |
| --- | --- | --- | --- | --- |
|  | **High** | **Medium** | **Low** |  |
| MACE | | | | |
| Model 1 | 1.00 (Ref) | 1.41 (1.35-1.48) | 2.01 (1.91-2.08) | <0.001 |
| Model 2 | 1.00 (Ref) | 1.13 (1.07-1.18) | 1.36 (1.30-1.42) | <0.001 |
| HF | | | | |
| Model 1 | 1.00 (Ref) | 1.63 (1.53-1.74) | 2.64 (2.49-2.80) | <0.001 |
| Model 2 | 1.00 (Ref) | 1.19 (1.12-1.28) | 1.56 (1.46-1.65) | <0.001 |
| MI | | | | |
| Model 1 | 1.00 (Ref) | 1.40 (1.32-1.47) | 1.89 (1.80-1.99) | <0.001 |
| Model 2 | 1.00 (Ref) | 1.09 (1.03-1.15) | 1.31 (1.25-1.38) | <0.001 |
| Stroke | | | | |
| Model 1 | 1.00 (Ref) | 1.44 (1.33-1.56) | 2.11 (1.96-2.27) | <0.001 |
| Model 2 | 1.00 (Ref) | 1.13 (1.04-1.23) | 1.43 (1.33-1.55) | <0.001 |

Model 1: unadjusted for covariates

Model 2: adjusted for age, sex, education, ethnicity, Townsend Deprivation Index, body mass index, smoking status, alcohol consumption, hypertension, type 2 diabetes, cancer and sleep duration.

**Supplementary Table 7. Sensitivity analysis of the associations between grip strength and MACE after additional adjustment for physical activity among patients with MASLD**

| **Outcome** | **Hazard ratio (95% CI)** | | | **P-value for trend** |
| --- | --- | --- | --- | --- |
|  | **High** | **Medium** | **Low** |  |
| MACE | | | | |
| No of events | 2426/30014 | 3211/29335 | 4506/29912 |  |
| Model 1 | 1.00 (Ref) | 1.40 (1.33-1.48) | 2.03 (1.93-2.13) | <0.001 |
| Model 2 | 1.00 (Ref) | 1.09 (1.03-1.15) | 1.35 (1.28-1.42) | <0.001 |
| Model 3 | 1.00 (Ref) | 1.09 (1.03-1.15) | 1.35 (1.28-1.42) | <0.001 |
| HF | | | | |
| No of events | 880/30014 | 1349/29335 | 2144/29912 |  |
| Model 1 | 1.00 (Ref) | 1.62 (1.49-1.77) | 2.64 (2.44-2.86) | <0.001 |
| Model 2 | 1.00 (Ref) | 1.19 (1.09-1.29) | 1.54 (1.42-1.67) | <0.001 |
| Model 3 | 1.00 (Ref) | 1.18 (1.09-1.29) | 1.52 (1.40-1.65) | <0.001 |
| MI | | | | |
| No of events | 1354/30014 | 1730/29335 | 2285/29912 |  |
| Model 1 | 1.00 (Ref) | 1.34 (1.25-1.44) | 1.80 (1.68-1.93) | <0.001 |
| Model 2 | 1.00 (Ref) | 1.07 (0.99-1.15) | 1.27 (1.18-1.36) | <0.001 |
| Model 3 | 1.00 (Ref) | 1.06 (0.99-1.14) | 1.26 (1.18-1.35) | <0.001 |
| Stroke | | | | |
| No of events | 614/30014 | 808/29335 | 1083/29912 |  |
| Model 1 | 1.00 (Ref) | 1.38 (1.25-1.54) | 1.88 (1.70-2.08) | <0.001 |
| Model 2 | 1.00 (Ref) | 1.08 (0.97-1.20) | 1.27 (1.15-1.41) | <0.001 |
| Model 3 | 1.00 (Ref) | 1.08 (0.97-1.20) | 1.27 (1.15-1.41) | <0.001 |

Model 1: unadjusted for covariates

Model 2: adjusted for age, sex, education, ethnicity, Townsend Deprivation Index, body mass index, smoking status, alcohol consumption, hypertension, type 2 diabetes, cancer and sleep duration

Model 3: adjusted for age, sex, education, ethnicity, Townsend Deprivation Index, body mass index, smoking status, alcohol consumption, hypertension, type 2 diabetes, cancer, sleep duration and physical activity.

**Supplementary Table 8. Sensitivity analysis of the associations between BMI-adjusted grip strength and MACE in patients with MASLD**

| **Outcome** | **Hazard ratio (95% CI)** | | | **P-value for trend** |
| --- | --- | --- | --- | --- |
|  | **High** | **Medium** | **Low** |  |
| MACE | 1.00 (Ref) | 1.28 (1.23-1.33) | 1.77 (1.67-1.88) | <0.001 |
| HF | 1.00 (Ref) | 1.52 (1.42-1.62) | 2.50 (2.29-2.73) | <0.001 |
| MI | 1.00 (Ref) | 1.19 (1.12-1.25) | 1.44 (1.32-1.56) | <0.001 |
| Stroke | 1.00 (Ref) | 1.25 (1.15-1.36) | 1.62 (1.44-1.82) | <0.001 |

Multivariable model was adjusted for age, sex, education level, ethnicity, Townsend Deprivation Index, smoking status, alcohol consumption, hypertension, type 2 diabetes, cancer, and sleep duration.
